# Root Hydraulic and Metabolomic Recovery Outpaces Stomatal Reopening in Rewatered Quinoa

**DOI:** 10.3390/plants15152280

**Published:** 2026-07-25

**Authors:** Flavia Dorochesi, Cesar Barrientos-Sanhueza, Marcos Roldán-Lazo, Romina Pedreschi, Italo F. Cuneo

**Affiliations:** 1 Facultad de Ciencias Agronómicas y de los Alimentos, Pontificia Universidad Católica de Valparaíso, Valparaíso 2340025, Chile; fl.dorochesi@pucv.cl (F.D.); marcosjr.ag@gmail.com (M.R.-L.); romina.pedreschi@pucv.cl (R.P.); 2 Millennium Institute Center for Genome Regulation, Santiago 8331150, Chile

**Keywords:** Lp_rOS_, drought–recovery dynamics, belowground hydraulics, halophyte resilience, root metabolomics

## Abstract

Drought research on quinoa has focused almost exclusively on the shoots, leaving the roots, the organ that first senses soil drying, largely unexamined, and its recovery dynamics are still poorly characterized. Here, we show that in the Chilean coastal quinoa ecotype AZ1, recovery from drought is governed belowground, and the root regains hydraulic and metabolomic competence well before the stomata reopen. After 72 h of soil drying, stomatal conductance (g_s_) decreased by 98%, whole-plant transpiration declined biphasically (~92% of the loss within the first two hours), and water potential decreased steeply at the soil–root interface (with soil and root water potential declining approximately 10- and 20-fold relative to well-watered plants), while the stem remained near-stable, pinpointing the root as the dominant hydraulic bottleneck. Twenty-four hours after rewatering, root system and whole-plant water potential, osmotic root hydraulic conductance (Lp_rOS_), root anatomy, and the polar metabolome were largely restored, yet g_s_ remained statistically indistinguishable from droughted plants. Strikingly, hydraulic recovery proceeded without rebuilding the osmotic sugar pool; instead, normalization of TCA-cycle intermediates points to an energy-powered and possible aquaporin-mediated transport route that bypasses still-suberized apoplastic barriers. Root system metabolomics, led by GABA and L-alanine, which overshot the control, tracked root rehydration but correlated negatively with g_s_, suggesting that nitrogen-rich solutes may act as candidate belowground cues restraining stomatal reopening. These findings suggest that the quinoa root system acts as a pacemaker for drought recovery.

## 1. Introduction

The root system is the principal interface between plants and the soil environment, securing anchorage and acquiring water and mineral nutrients [1]. Within the soil–plant–atmosphere continuum (SPAC), water movement is driven by a water potential gradient across successive hydraulic resistances, flowing from the bulk soil to the stomatal cavity [2,3]. Although traditionally viewed as a passive segment of this continuum, an expanding body of evidence has repositioned the root system as the primary integrator of plant water status regulation and the dominant control point for stomatal closure under progressive soil drying [4]. Whereas the classical leaf-centric paradigm attributed stomatal closure to declines in leaf water potential and xylem hydraulic conductance [5,6], subsequent studies have demonstrated that closure frequently precedes xylem failure thresholds, implicating the soil–root interface as the earliest bottleneck constraining transpiration [7,8,9]. Reference [10] formalized this by proposing that conductance loss at the soil–root interface, rather than xylem vulnerability, is the primary trigger of stomatal regulation under drought conditions. This framework has gained empirical traction across functionally diverse species, including olive [11], tomato [12], wheat [9], and grapevine [13], and has been consolidated as a unifying principle of crop hydraulic behavior in water-limited environments [14], repositioning the root as the primary regulator of stomatal aperture during soil drying rather than a passive conduit.

The mechanism by which roots exert hydraulic control under drought conditions relies on complementary processes operating in parallel. On the one hand, root hydraulic conductivity is dynamically tuned through aquaporin gating and abundance at the plasma membrane [1,15]. In contrast, the radial pathway of water transport is concurrently reshaped by plastic remodeling of the endodermis, particularly through the deposition of apoplastic barriers, such as suberin lamellae and Casparian strips, which constrain apoplastic flow [16]. Collectively, these adjustments modulate water uptake but impose a substantial biosynthetic and energetic cost [17] that can only be met by a concomitant reconfiguration of the primary root metabolism [18]. Among these metabolic responses, the accumulation of compatible osmolytes (including soluble carbohydrate pools and low-molecular-weight nitrogenous solutes) is widely recognized as the primary driver of osmotic adjustment, lowering the tissue osmotic potential to sustain water uptake and turgor as the soil water potential declines [19,20]. Among these, soluble sugars, such as glucose, fructose, and sucrose, are dynamically modulated under water deficit, acting in a dual capacity: as compatible solutes that lower the cellular osmotic potential and sustain turgor-driven water uptake, and as respiratory substrates that provide the energy required to maintain active membrane transport under carbon-limiting conditions [21,22,23]. Beyond these osmotic and bioenergetic roles, a fraction of these sugars is redirected to play an additional protective role against oxidative damage during drought [24,25,26]. Similar to soluble sugars, nitrogen-rich solutes (such as GABA and amino acids) and tricarboxylic acid (TCA) cycle intermediates function beyond basic osmotic adjustment; under stress, they act in a comparable manner to regulate cytosolic pH, maintain redox homeostasis, and sustain cellular energy status [27,28]. Taken together, these metabolic adjustments provide the osmotic, protective, and energetic currency that underpins the hydraulic and anatomical remodeling of the root outlined above [29], thereby positioning root primary metabolism as a central determinant of water-uptake capacity during both drought and recovery. Despite these advances, the temporal coordination of these three components (hydraulic, structural, and metabolic) within the root system and whether such coordination is preserved after post-drought recovery remains largely unresolved.

Among the halophytic crops of high agronomic value, quinoa (*Chenopodium quinoa* Willd.) has emerged as a valuable model for stress resilience owing to its exceptional combination of drought and salinity tolerance, agronomic plasticity, and nutritional value [30,31,32,33]. Quinoa is a facultative halophyte of the Amaranthaceae whose prolonged domestication across the Andes, from the altiplano to sea level, has generated five recognized ecotypes (highland, inter-Andean valley, salares, Yungas, and coastal lowlands) and a correspondingly broad intraspecific variability [34]. Chilean germplasm is genetically and ecophysiologically among the most diverse within this range [35,36], and tolerance to drought and salinity is strongly genotype-dependent, generally tracking the aridity of the habitat of origin [37,38,39]. However, drought research on quinoa has focused primarily on shoot-level descriptors (such as leaf gas exchange, photosynthetic performance, and leaf water relations), leaving root systems significantly understudied [40,41,42,43,44]. Although belowground traits have been addressed in this species, attention has been directed mainly at root growth and architecture rather than root function. In particular, root elongation and root-to-shoot growth and allocation have been used to discriminate tolerant from sensitive Chilean lowland genotypes [37]. Root foraging capacity has been related to root system architecture and ontogeny in Andean *Chenopodium* species [45]. Coastal and salares landraces have been shown to differ in root length and root biomass allocation under stress [38]. Roots have been reported to be more susceptible than shoots to ion toxicity, with reductions in root length, surface area, and volume [46]. In contrast, root water transport, the anatomy of the radial pathway, and root primary metabolism during soil drying and recovery remain comparatively unexplored. Early physiological studies have demonstrated that quinoa exhibits strong stomatal sensitivity to soil drying, which is only partially explained by xylem-derived chemical signals [39]. Furthermore, indirect evidence suggests that belowground water transport is restricted before substantial declines in the leaf water status occur [40]. Although recent studies on coastal quinoa under salinity have shown tightly coordinated root–shoot hydraulic adjustments compatible with the soil–root paradigm of stomatal regulation [30], and although comparative work has established that coastal lowland landraces, native to sea-level habitats with higher rainfall and non-saline soils, deploy stress strategies that differ markedly from those of altiplano and salares germplasm [37,38,47,48,49], an integrated, mechanistic characterization of the hydraulic, anatomical, and metabolic root responses to progressive soil drying remains to be established. Notably, the recovery dynamics of these root-level processes have not been explored in this species. The rewatering phase offers a critical window into plant–water regulation, as the capacity to restore physiological function after stress relief largely determines crop performance under intermittent droughts projected for current climate scenarios [50,51]. This is particularly pertinent for quinoa, since climate projections anticipate less frequent but more intense rainfall events that lengthen the intervening periods of dry soil, a regime under which crop performance depends as much on the capacity to recover as on the capacity to endure stress [34]. These recovery trajectories also reveal whether stress-induced modifications are reversible or persistent, a critical distinction that the drought phase alone cannot expose [52,53]. Ultimately, whether root-level restoration proceeds concomitantly with stomatal reopening or follows a decoupled trajectory upon rewatering remains poorly understood.

Despite mounting evidence that belowground hydraulics govern stomatal behavior during soil drying, the temporal coordination between root and shoot recovery upon rewatering remains unresolved, particularly in halophytic crops adapted to water-limited environments such as quinoa. Whether the root regains hydraulic competence in synchrony with or ahead of the resumption of g_s_ and the metabolic basis of any such asymmetry have not been mechanistically addressed. In this study, we hypothesized that in coastal quinoa AZ1, the root governs stomatal behavior during drought recovery through both its hydraulic and metabolomic states. Upon rewatering, root water status and Lp_rOS_ are restored ahead of stomatal reopening, with g_s_ remaining constrained by residual root-derived cues, both hydraulic and metabolomic, until belowground recovery is fully consolidated. Here, we examined the drought–recovery dynamics of quinoa by integrating gas exchange, water potential across the SPAC, Lp_rOS_, root anatomy, and root polar metabolite profiling.

## 2. Materials and Methods

### 2.1. Plant Material

The coastal ecotype AZ1 of Chenopodium quinoa was used in this study. Seeds were sourced from the coastal locality of Pichilemu in the Libertador Bernardo O’Higgins Region [54]. AZ1 is a coastal lowland ecotype, and the germplasm from this locality has been characterized as belonging to the coastal lowland group, which grows at sea level on marine terraces with periodically elevated soil salt content and is genetically distinct from the salares and highland germplasm [35,36,38]. This ecotype was selected because it combines documented tolerance to salinity and drought with an agronomically relevant sea-level habitat, making it a suitable model for examining belowground recovery [34,37]. Seed sterilization and germination were performed following the protocol described by [30]. Seedlings were then transplanted into 1.0 L plastic pots filled with coarse quartz sand (56% with a particle size of 0.5 mm, 38% of 0.25 mm, 5% of 0.15 mm, and 1% of 0.053 mm). The soil had a bulk density of 0.771 g cm^−3^ and a volumetric field capacity of 11.6%, determined by saturating the pots and allowing free drainage for 1 h [30,55,56]. Coarse quartz sand was used as the growth medium, providing high porosity and reduced water retention [57]. The transplanted quinoa plants were maintained in a greenhouse for five weeks to promote plant development under controlled environmental conditions in the greenhouse, with temperature maintained between 25 and 28 °C and relative humidity at approximately 35%. The experiment was conducted at the greenhouse facilities of the Pontificia Universidad Católica de Valparaiso, Quillota, Chile (coordinates: 32°53′ S, 71°12′ W), during January–March 2026). Natural light (PAR of 500–1100 μmol photons m^−2^ s^−1^) was supplemented with 600 W metal-halide bulbs (PIRANHA, Jiangsu, China, PAR of 400–600 μmol photons m^−2^ s^−1^), providing a photoperiod of 16 h [58], and plants were watered twice a day with a supplemented irrigation solution to reach 95% field capacity (i.e., macronutrients and micronutrients; [59]).

### 2.2. Plant Stress Conditions

The experimental design included three treatments: (i) a well-watered control, (ii) a drought treatment in which irrigation was withheld for three days, and (iii) a recovery treatment, in which the plants were re-irrigated to near field capacity after the drought period and then allowed to recover for 24 h. In this low-retention quartz-sand soil, three days of water withholding were sufficient to drive plants into severe water stress ($\Psi_{stem}\leq -1.2\;MPa$; see Section 3.2), which justified the duration of the drought period. A total of 135 uniform plants (45 per treatment) were randomly selected. Because all measurements required destructive harvesting, independent sets of plants were assigned to each treatment and sampling point, where the drought and recovery treatments corresponded to different plants sampled sequentially and did not belong to the same pots measured repeatedly. At the beginning of the experiment, all plants were irrigated with 100 mL of irrigation solution to reach approximately 95% field capacity. Thereafter, irrigation was suspended only in the drought and recovery treatments, whereas the control plants were maintained at 95% field capacity through regular irrigation. The experiment was conducted under the controlled conditions described in the previous section. At the time of sampling, the plastic pots were opened, and the shoots and roots were gently rinsed with filtered water to remove any soil. The relative water content (RWC) of the whole plants, shoots, and roots was determined from the fresh weight (FW), turgid weight (TW), and dry weight (DW), where FW was recorded immediately after harvest, TW after full rehydration of the tissue in distilled water at 4 °C for 24 h, and DW after oven-drying at 70 °C to a constant mass (approximately three days), and calculated as follows:

RWC (%) = ((FW − DW)/(TW − DW)) × 100(1)

### 2.3. Transpiration Rate Measurements

The transpiration rate was determined gravimetrically using plants maintained at 95% field capacity. Prior to measurement, each pot was completely sealed with Parafilm and plastic bags, leaving only the aerial portion of the plant exposed to the atmosphere, thereby minimizing water loss from the substrate. The pots were individually placed on a Sartorius precision balance (Entris II, Sartorius, Göttingen, Germany), and the system weight was recorded every 5 min for 72 h. Under these conditions, changes in weight over time were assumed to reflect water loss via plant transpiration. The transpiration rate was calculated from the slope of the weight loss over time.

### 2.4. Measurements of Water Potential

The water potentials of the soil, roots, stems, and leaf tissues were determined using a dew point hygrometer (WP4C, METER Group, Pullman, WA, USA) operated in precise mode. The WP4C measures the total water potential, which reflects the combined contributions of matric and osmotic. Soil and root samples were collected from the middle portion of the pot as close as possible to the root system. Stem samples were collected from the middle section of the plant, and leaf samples were collected from leaves located in the same zone. All samples were placed immediately into individual sample cups to minimize water loss prior to analysis, and measurements were recorded once the readings stabilized. All water potential measurements were performed at midday (11:00–13:00 h), concurrently with gas-exchange measurements, to capture the period of maximum evapotranspiration demand and treatment differentiation and not under predawn equilibrium.

In parallel, leaf (Ψ_leaf_) and stem water potentials (Ψ_stem_) were measured using a Scholander pressure chamber (PMS model 1505D; Albany, OR, USA). For Ψ_stem_ determination, leaves were enclosed in an aluminized plastic bag for at least 20 min, following the method described by [60], to minimize transpiration and allow equilibration with the stem xylem. For Ψ_leaf_ determination, leaves were enclosed in a Ziploc^®^ bag prior to excision to minimize water loss before the measurement. Leaves were then excised at the base of the petiole and immediately placed in a pressure chamber for measurement. Two leaves per plant were selected for each of the measurements. Stress levels were defined as follows: low water stress (Ψ_stem_ ≥ −0.6 MPa), mild water stress (−0.6 MPa > Ψ_stem_ > −1.2 MPa), and severe water stress (Ψ_stem_ ≤ −1.2 MPa) [61].

### 2.5. Stomatal Conductance

Stomatal conductance (g_s_), leaf transpiration (*E*), and leaf vapor pressure deficit (VPD_leaf_) were measured using a LI-COR porometer/fluorometer (LI-600PF, LI-COR Biosciences, Lincoln, NE, USA). Measurements were performed on the same leaves as previously described for leaf and stem water potential, between 11:00 and 13:00 h under the same greenhouse conditions detailed in Section 2.1 (see above). Stomatal conductance was measured on two fully expanded leaves located in the middle section of each plant.

### 2.6. Measurements of Osmotic Root Hydraulic Conductance

An intact root system was used to measure the osmotic root hydraulic conductance (Lp_rOS_). Plants were carefully removed from the pots, and the root systems were washed to remove sand. The shoots were then excised under water to prevent xylem embolism. A hose with an internal diameter of 7.5 mm was connected to the cut stem and used as an interface between the stem and capillary tube with an internal diameter of 0.05 mm. Both were connected using a microfluidic adapter, and all junctions were sealed with Parafilm. Prior to the experiment, the system was checked for any leaks. The capillary was half-filled with distilled water to ensure that the cut stem was never exposed to air. The root systems were equilibrated in distilled water for 1 h [57]. Subsequently, they were subjected to mannitol solutions with osmotic potentials of 0, −0.125, −0.25, and −0.5 MPa, applied in decreasing order. Before each measurement, the root system was allowed to equilibrate in each solution for 30 min, after which the meniscus displacement in the capillary was monitored every 4 min for 24 min using a digital camera. The changes in volume (V) were calculated for each osmotic step based on the displacement of the meniscus in the capillary tube. The volumetric flow rate (Q; m^3^ s^−1^) was calculated as the slope of the linear regression between the cumulative volume (m^3^) and time (s) for each osmotic step. Lp_rOS_ was calculated using the following equation:

Lp_rOS_ = (Q/ΔΨ)(1/FW) (2)

where Q/ΔΨ (m^3^ s^−1^ MPa^−1^) corresponds to the slope of the relationship between the volumetric flow rate (Q) and the applied osmotic gradient (ΔΨ; Supplementary Figure S1) [59,62], and FW is the fresh weight of the root system (g). Thus, Lp_rOS_ was expressed as m^3^ s^−1^ MPa^−1^ g FW^−1^ [63].

### 2.7. Fluorescence Microscopy

To examine the impact of drought on fine root cortex integrity and structural alterations, free-hand cross-sections were made using a razor blade in the root maturation and absorptive (active root hair development) zones (20–70 mm from the tip). To visualize suberin and the Casparian strip, root sections were stained following the methodology described by [57,63]. The stained root sections were mounted on glass slides and observed under UV/violet fluorescent light (excitation filter, 355–425 nm; dichromatic mirror, 455 nm; and suppression filter, 470 nm) using a Leica DMIL LED inverted microscope (Leica Microsystems, Wetzlar, Germany).

Images were captured using a digital camera (Leica MC170HD; Leica Microsystems, Wetzlar, Germany). The Casparian and suberin bands appear yellow, while the living tissue (cell walls with β-glucans) is sky-blue.

### 2.8. Root Polar Metabolite Extraction and Analysis

Roots of quinoa plants were collected from the middle section of the pot. Samples were washed with distilled water to remove adhering sand particles and rapidly but carefully blotted dry before being immediately frozen in liquid nitrogen. The frozen material was then ground, and 0.5 g of each sample was freeze-dried again. Extraction, derivatization, and analysis of polar metabolites were performed as described by [64]. However, absolute quantification was not performed, which we acknowledge as a limitation of this study. Metabolite profiling was performed using an Agilent 7890B gas chromatograph (Agilent Technologies, Santa Clara, CA, USA) coupled to a PAL3 autosampler and a 5977A single-quadrupole mass spectrometer equipped with an electron impact ionization source (EI 350). Separation was achieved using an HP-5-MS Ultra Inert capillary column (30 m × 0.25 mm, 0.25 µm; Agilent Technologies, Santa Clara, CA, USA). The analysis focused on primary polar metabolites, including sugars, amino acids, and organic acids in the roots. Metabolites were identified based on retention time and mass spectral data using a custom-built library of commercial standards and the NIST14 database, and data were processed with MassHunter Quantitative software 12.0 (Agilent Technologies, Santa Clara, CA, USA). The metabolite abundances were expressed as relative abundances.

Differential abundance analysis of the metabolomic data was performed using MetaboAnalyst v6.0. The data were auto-scaled by mean centering and dividing each variable by its standard deviation prior to statistical analysis. Differentially abundant metabolites (DAMs) among the treatments were identified using one-way ANOVA, and significance was determined using a false discovery rate (FDR) threshold of 0.05. Heatmaps and multivariate analyses, including principal component analysis (PCA) and partial least squares discriminant analysis (PLS-DA), were generated in R using an auto-scaled dataset exported from MetaboAnalyst.

### 2.9. Data Analysis

Data were analyzed using R v4.4.2 (R Foundation for Statistical Computing, Vienna, Austria). Data normality was checked using the Shapiro–Wilk test and homogeneity of variances using Levene’s test. When the assumptions were violated, the data were suitably transformed. Treatment effects were evaluated using one-way ANOVA, followed by Tukey’s HSD test. Figures were generated using the ggplot2 package, whereas metabolomic multivariate analyses (PCA and PLS-DA) and heatmaps were produced using MetaboAnalyst v6.0. 

## 3. Results

### 3.1. Stomatal Conductance and Whole-Plant Water Loss Dynamics

**Figure 1:**
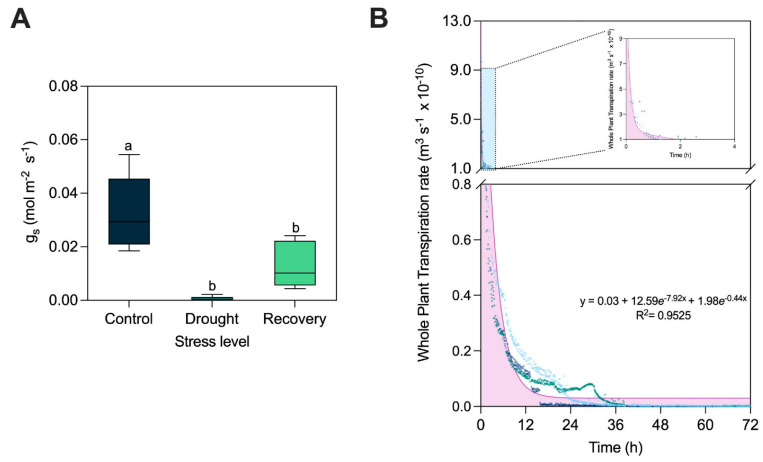
Stomatal conductance and whole-plant water loss dynamics in coastal quinoa AZ1 under drought stress and during recovery. (**A**) Stomatal conductance (g_s_) measured before stress imposition (control), after 72 h of drought, and after 24 h of recovery following rehydration. Data are presented as mean ± SE (*n* = 5). Different letters indicate significant differences among the treatments (Tukey’s HSD test, *p* < 0.05). (**B**) Whole-plant transpiration rate measured over 72 h of drought (*n* = 3). The individual plant behaviors are indicated in blue tones. Water loss was modeled using a bi-exponential decay function (R^2^ = 0.95), represented by a purple fitted curve. The upper inset shows an expanded view of the first 4 h of the dry-down period.

Stomatal conductance (g_s_) was measured at the start of the experiment, after 72 h of drought, and after 24 h of recovery following rewatering, and it differed significantly among the treatments (*p* = 7.01 × 10^−4^). Drought drove a sharp decline in g_s_ and lowered the mean by 98%, whereas after 24 h of rewatering, g_s_ was still almost 60% lower than that in the well-watered treatment, with non-statistical differences (Figure 1A), which shows that 24 h was not enough for full functional restoration of stomatal activity. Leaf-level transpiration (*E*) followed the same trend as g_s_, whereas VPD varied comparatively little among treatments, with drought plants holding intermediate values between control and recovery (Supplementary Table S1).

At the whole-plant level, the transpiration rate over the 72 h dry-down period was captured accurately by a bi-exponential decay function (R^2^ = 0.95; Figure 1B). The model resolved two phases of water loss: a rapid initial drop during the first 2 h, as shown in the inset, during which the plants lost roughly 92% of the total water reduction recorded over 72 h, followed by a slower and more gradual decline as drought progressed. This biphasic pattern matched the rapid fall in g_s_ observed under drought conditions.

### 3.2. Plant Water Status

**Figure 2:**
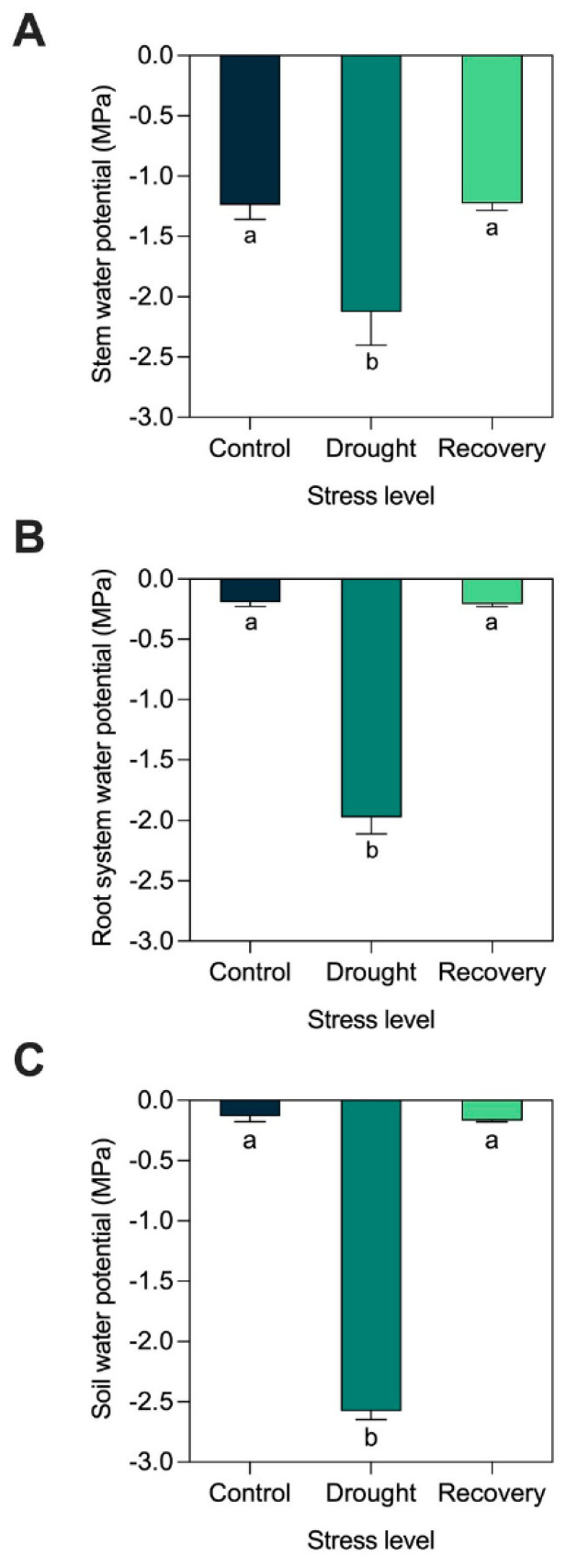
Water potential dynamics across the soil–plant continuum during drought and rewatering. Water potential was determined using a WP4C dewpoint potentiometer in stems (**A**), roots (**B**), and soil (**C**) under control, drought, and recovery conditions. Bars represent the mean ± SE (*n* = 5). Different letters indicate significant differences among treatments (Tukey’s HSD test, *p* < 0.05).

The water status of coastal quinoa AZ1 was strongly affected by drought but fully recovered 24 h after rewatering (Figure 2). Drought treatment induced a marked decline in water potential across the soil–plant continuum (*p* < 0.001 for all components), with the most pronounced decreases observed in the soil (almost 20-fold) and roots (10-fold), and a more moderate decrease in the stems (almost 2-fold). After 24 h of rewatering, the water potential returned to values that were not significantly different from those of the control plants. This full hydraulic recovery preceded the restoration of the stomatal conductance (Figure 1A). The same pattern was observed when plant water potential was assessed using the Scholander pressure chamber (Supplementary Figure S2), which supports the WP4C measurements and confirms that the drought-induced reductions in plant water potential were largely reversible within the recovery period.

This recovery was also observed in the relative water content (RWC). Drought significantly lowered RWC; however, values returned to control-like levels of approximately 97% across all tissues after rewatering (Supplementary Table S2). The physiological resilience of AZ1 was confirmed visually by plant phenotype, as droughted plants showed severe wilting and loss of whole-plant turgor, whereas rewatered plants regained their upright position and leaf turgidity (Supplementary Figure S3).

### 3.3. Osmotic Root Hydraulic Conductance

**Figure 3:**
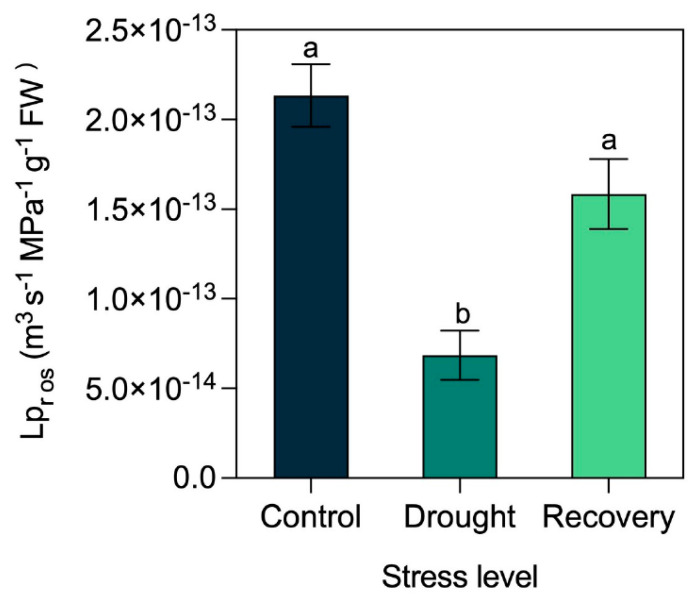
Osmotic root hydraulic conductance (Lp_rOS_) of coastal quinoa AZ1 under control, drought, and recovery conditions. Data are presented as mean ± SE (*n* = 4). Different letters indicate significant differences among the treatments (Tukey’s HSD test, *p* < 0.05).

Osmotic root hydraulic conductance (Lp_rOS_) differed significantly among the treatments (*p* = 2.70 × 10^−3^). After 72 h of drought, a marked reduction in Lp_rOS_ was observed, which decreased by 68% relative to the control (Figure 3). Following rewatering, Lp_rOS_ showed a substantial recovery, returning to values comparable to the control, although it remained 26% lower than the control.

### 3.4. Root Anatomy

**Figure 4:**
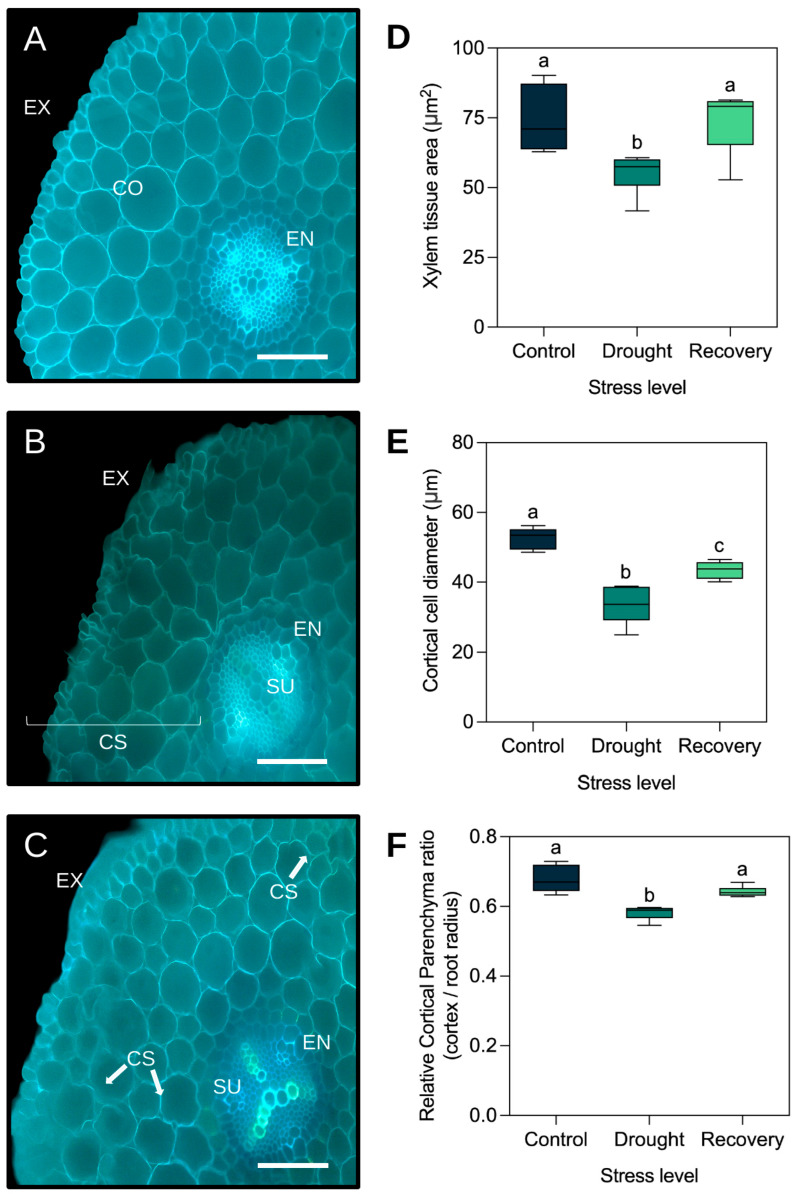
Root anatomy and quantitative analysis of coastal quinoa AZ1 under drought and recovery conditions. Representative fluorescence microscopy images of freehand root cross-sections of control (**A**), drought (**B**), and recovery (**C**) plants are shown. (**D**) Xylem tissue area, (**E**) cortical cell diameter, and (**F**) relative cortical parenchyma ratio (cortex/root radius). EX, exodermis; CO, cortex; EN, endodermis; SU, suberization; CS, cellular shrinkage. White bar = 100 μm. Boxplots represent the median and interquartile range (*n* = 8). Different letters indicate significant differences among the treatments (Tukey’s HSD test, *p* < 0.05).

Fluorescence microscopy of root cross-sections revealed distinct morphological changes in response to drought and subsequent recovery. Under well-watered conditions, the cortical cells exhibited a normal spherical shape when fully turgid, with intact cell walls and no signs of tissue damage, and the cyan fluorescence observed throughout the cortex was related to the presence of living tissue (Figure 4A). Under drought conditions, marked cellular shrinkage (CS) was observed throughout the exodermis, cortical parenchyma, and endodermis. This was accompanied by visible cell wall folding, consistent with severe dehydration, and suberization of the endodermis and xylem (Figure 4B). Following rewatering, the cortical cells partially recovered their turgid appearance, although some degree of cellular shrinkage persisted in the cortex (Figure 4C). Suberization was also observed in the xylem vessels of recovery plants.

Quantitative analysis confirmed this observation. The xylem tissue area differed significantly among treatments (*p* = 7.4 × 10^−3^), and decreased by 25% under drought relative to the control, whereas recovery plants showed near-complete restoration, with values only 0.50% lower than the control, and no significant differences among them (Figure 4D). Cortical cell diameter was significantly affected (*p* = 1.11 × 10^−6^), declining by 14% under drought and remaining 5% below well-watered after rewatering (Figure 4E), and all three conditions differed significantly from each other. Similarly, the relative cortical parenchyma ratio differed significantly among treatments (*p* = 4.98 × 10^−5^), decreasing by 36% under drought and returning to levels statistically similar to the control following rewatering, despite being 17% lower (Figure 4F).

### 3.5. Metabolomic Analysis

**Figure 5:**
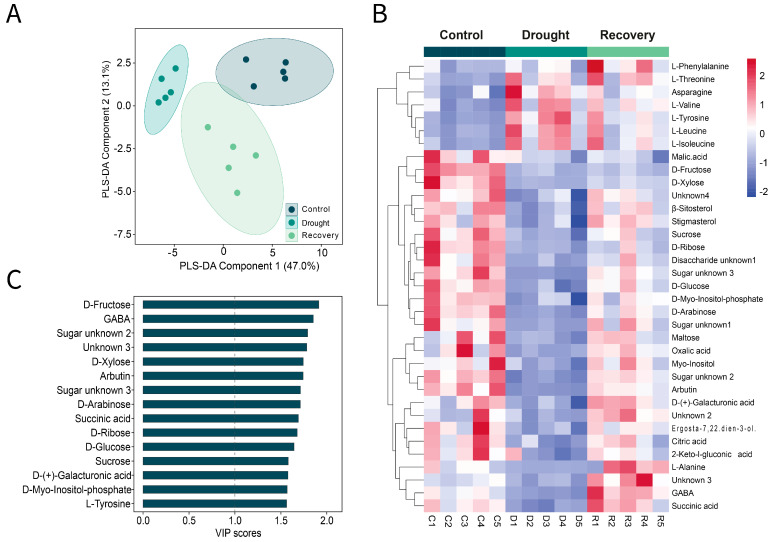
Metabolomic profiling of coastal quinoa AZ1 roots under control, drought, and recovery conditions. (**A**) Partial least squares discriminant analysis (PLS-DA) score plot illustrating the clustering of biological replicates across treatments based on the relative abundance of identified metabolites. The numbers in parentheses indicate the percentage of variance explained by the first two components. (**B**) Heatmap visualizing the z-score-normalized relative abundances of the 35 statistically significant metabolites identified across treatments. The color scale represents the relative enrichment (red) or depletion (blue) of each metabolite. (**C**) Variable importance in projection (VIP) scores derived from the PLS-DA model, highlighting metabolites with VIP > 1.0 as the most significant contributors to treatment separation.

To evaluate the metabolomic response of the coastal quinoa AZ1 root system to drought and recovery, PLS-DA was performed (Figure 5A). The score plot revealed a separation among treatments, with samples from each group forming distinct clusters, primarily driven by principal components 1 and 2 (PC1 and PC2), which explained 47% and 13% of the total variance, respectively. Of the 49 metabolites identified, 35 changed significantly among the treatments (ANOVA *p* < 0.05) (Supplementary Table S3). A color-scaled heatmap was constructed using these 35 metabolites (Figure 5B), where the color scale represents the relative enrichment or depletion across treatments. From this set, 15 metabolites were selected for detailed analysis based on their VIP scores (>1) and biological relevance, encompassing sugars, organic acids, and amino acids (Figure 5C and Figure S4). D-fructose and GABA exhibited the highest VIP scores in the PLS-DA model (Figure 5C), with GABA also showing one of the lowest *p*-values among all identified metabolites (*p* = 2.68 × 10^−5^).

Overall, drought stress induced a broad metabolomic depression, with most carbohydrates and organic acids declining markedly under water deficit (Figure 5B; Supplementary Figure S4). Monosaccharides (D-fructose, D-xylose, D-ribose, D-glucose, and D-arabinose) decreased by 54–92% under drought compared to well-watered conditions, consistent with the severe stomatal closure observed in the plants. Among amino acids, L-alanine and GABA also decreased significantly under drought by 87% and 75%, respectively. In contrast, L-tyrosine and L-leucine increased 2.6- and 2.5-fold, respectively, relative to the control, while L-phenylalanine did not show significant changes during the stress period. Both TCA cycle intermediates, citric acid and succinic acid, also decreased by 67% and 60%, respectively, during drought. The osmolytes and signaling molecules myo-inositol and D-myo-inositol-phosphate also declined significantly under drought stress, with reductions of 54% and 43%, respectively. Additionally, sucrose, arbutin, and D-(+)-galacturonic acid levels decreased by 33%, 90%, and 39%, respectively, under drought conditions.

Following rewatering, the metabolites showed four distinct recovery patterns (Supplementary Figure S4). The first group, comprising D-glucose, myo-inositol, D-myo-inositol-phosphate, D-(+)-galacturonic acid, citric acid, succinic acid, and arbutin, returned to levels statistically similar to the control, indicating complete metabolomic restoration. In contrast, D-fructose, D-xylose, D-ribose, sucrose, L-tyrosine, and L-leucine levels were comparable to those observed under drought. D-arabinose showed an intermediate response, increasing 2.96-fold relative to the drought treatment but remaining significantly lower than the control values. Notably, GABA, L-alanine, and L-phenylalanine surpassed control levels during recovery, reaching 1.59-, 2.15-, and 2.09-fold the control values, respectively (Figure 5B; Supplementary Figure S4).

## 4. Discussion

**Figure 6:**
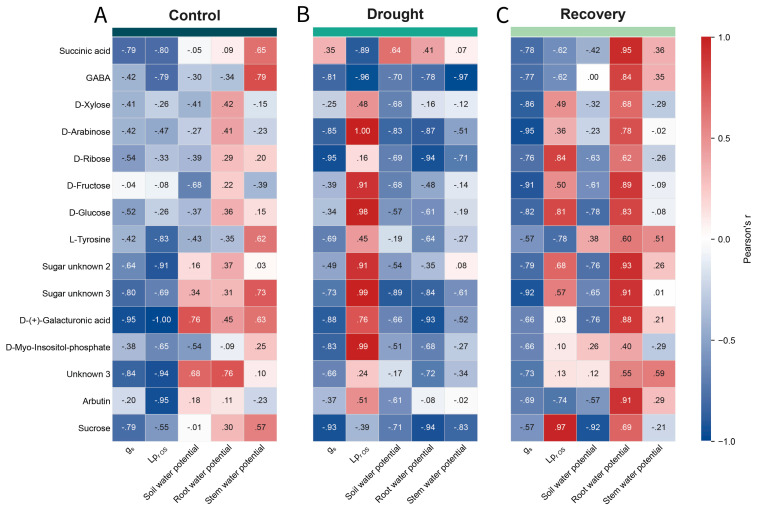
Pearson correlation matrices between root metabolites and physiological variables in coastal quinoa AZ1 under control (**A**), drought (**B**), and recovery (**C**) treatment. The physiological variables assessed were stomatal conductance (g_s_), root osmotic hydraulic conductance (Lp_rOS_), soil water potential (Ψ soil), root water potential, and stem water potential. The color scale represents the Pearson correlation coefficient (r) ranging from −1.0 (deep blue, perfect negative correlation) to +1.0 (deep red, perfect positive correlation). Only statistically significant correlations are shown (*p* < 0.05).

Our findings show that during the acute phase of drought recovery, the return of stomatal conductance in coastal quinoa AZ1 is mechanistically uncoupled from the restoration of whole-plant water status, since the belowground hydraulic and metabolomic machinery regains function well ahead of the foliar gas exchange apparatus. After 24 h of rewatering, the water potential was returned to control values across the entire SPAC (Figure 2; Supplementary Figure S2), and this hydraulic recovery was mirrored by a near-complete restoration of osmotic root hydraulic conductance (Lp_rOS_; Figure 3), by the apparent rehydration of cortical cells and partial recovery of xylem tissue area (Figure 4), and by resetting the root polar metabolome (Figure 5B). In contrast, stomatal conductance (g_s_) remained severely depressed and statistically indistinguishable from that of drought-stressed plants (Figure 1A), indicating that the physical and metabolomic recovery of the root system occurred at a markedly faster kinetic scale than the resumption of transpiration. This asymmetry is difficult to reconcile with the classical leaf-centric view, which holds that stomatal aperture is set by leaf or stem water status and by the instantaneous equilibration of guard cell turgor with the bulk leaf water potential [5]. The recovery Pearson correlation matrix demonstrated that root system polar metabolites were positively correlated with the recovery of root water potential but were strongly negatively correlated with g_s_ (Figure 6C), which indicates that the belowground compartment actively tracks rehydration while the stomatal complex remains restrained. We read this residual suppression of g_s_, which outlasts the structural and hydraulic recovery of the root system, as evidence of a conservative, threshold-like control point that enforces a cautious water-use strategy once stress is relieved, whether imposed by persistent root-to-shoot chemical signaling [47,51], residual hydraulic cues [11], or a leaf-autonomous hydraulic–hormonal set point in which guard cell aperture is affected by foliar ABA dynamics rather than by root-derived cues [65].

To resolve this asymmetry, we first examined the drought constraints that roots must overcome. At the whole-plant level, the transpiration record showed how quickly the coastal ecotype AZ1 restricts water loss during soil drying, as approximately 92% of the total decline in transpiration rate occurred within the first 2 h (Figure 1B). This rapid drop ran in parallel with near-total suppression of g_s_ (Figure 1A) and matched the high stomatal sensitivity to early water deficit reported for quinoa [47]. The water potential profile pinpoints the location of the main hydraulic resistance behind this phenomenon. The steep decline in soil and root water potentials, set against a comparatively smaller decline in stem water potential (Figure 2), shows that the dominant hydraulic resistance lies at the soil–root interface rather than within the shoot xylem [10,11]. The force of this belowground stress was clear at the tissue level, where fine roots showed pronounced cellular shrinkage across the exodermis, cortical parenchyma, and endodermis, together with cell wall folding and suberization of the endodermis and xylem (Figure 4B). In drought-susceptible species, cortical collapse and rigid suberization usually trigger an irreversible loss of root water transport through mechanical failure and air gaps at the soil–root interface [57,61]. However, coastal quinoa AZ1 overrode this structural constraint. Such resilience is consistent with the reported plasticity of the quinoa root system, which develops a deep and ramified architecture, whose foraging capacity depends on root system architecture and ontogeny, and with evidence that root length and root biomass allocation are among the traits that most clearly separate quinoa genotypes according to the aridity of their habitat of origin [34,37,38,45]. Although cortical cell diameter did not fully reset and remained approximately 5% below the control, both xylem tissue area and cortical parenchyma ratio recovered to control-equivalent values within 24 h (Figure 4D–F), while osmotic root system hydraulic conductance (Lp_rOS_) rebounded to values statistically comparable to the control (Figure 3). Lp_rOS_ was assessed under osmotic gradients, a driving force that preferentially moves water across semipermeable membranes and, therefore, reports the cell-to-cell pathway [57,65,66]. The substantial recovery of Lp_rOS_ observed here, despite persistent endodermal suberization and incomplete cortical re-expansion (Figure 3 and Figure 4), provides evidence consistent with this membrane-controlled route partially regaining competence, whereas the apoplastic pathway remained physically constrained [16,55]. This indicates a dynamic metabolic adjustment of membrane water permeability, possibly through the increased abundance and activity of plasma membrane intrinsic proteins. Enhanced PIP expression in quinoa roots under drought conditions has been previously reported [1,15,67]. Taken together, these findings suggest that transcellular transport, possibly aquaporin-mediated, can act as a compensatory mechanism that bypasses persistent apoplastic constraints, restores Lp_rOS_, and sustains plant turgor before the stomata reopen. This reconfiguration of the radial water transport pathway may be a central determinant of the hydraulic resilience underlying the rapid recovery of quinoa after rewatering.

The clear spatial separation in the PLS-DA score plot, where the first two components jointly captured 60% of the total metabolomic variance (Figure 5A), indicates highly concerted metabolomic reprogramming in the quinoa root system across the drought recovery treatments. In line with the coordinated metabolomic transitions documented during drought and rewatering cycles in diverse crops, this reprogramming was driven by D-fructose and GABA, which were the highest VIP discriminants (Figure 5C; [35,68,69]). A striking feature of the drought metabolome was the depletion, rather than accumulation, of root monosaccharides such as D-fructose, D-glucose, D-xylose, D-arabinose, and D-ribose, which runs opposite to the canonical role of soluble sugars as accumulating osmolytes (Figure 5C; Supplementary Figure S4; [19,20,22]). Rather than a failure in osmotic regulation, this depletion reflects a limitation of monosaccharides [70]. Early stomatal closure under drought conditions (Figure 1A,B) halts photosynthetic carbon gain and cuts off the shoot-derived supply to the root system [50,71]. This depletion most likely reflects the redirection of available carbohydrates toward the energetic and biosynthetic demands of root system acclimation, in particular, the deposition of apoplastic barriers through endodermal and xylem suberization (Figure 4) and the sustained respiratory cost of cellular maintenance under stress [17,23]. Therefore, the decline in the root system carbohydrate pool in droughted AZ1 did not serve as a substrate for osmolyte accumulation. It marks a sink that is starved of carbon, deprived of photo-assimilate by stomatal closure, and drawn down by the energetic cost of acclimation. A comparable interpretation has been advanced for quinoa under salinity, where reduced stomatal conductance and the attendant decline in photosynthetic rate were invoked to explain the redirection of carbon towards protective more than productive metabolism, and where the resulting compromise between stress resistance and biomass accumulation has been described as a growth-tolerance trade-off [38,39,72].

Beyond carbohydrates, drought reconfigured root nitrogen metabolism in a bifurcated manner. GABA and L-alanine collapsed (Supplementary Figure S4; Supplementary Table S3), whereas the branched-chain amino acids L-leucine, L-isoleucine, and L-valine, the aromatic amino acids L-tyrosine and L-threonine, and asparagine accumulated markedly (Figure 5B; Supplementary Table S3). These two trajectories have distinct metabolic origins. GABA and L-alanine are synthesized from central carbon metabolism, L-alanine from glycolytic pyruvate and GABA from TCA-derived glutamate; therefore, their decline most likely reflects the precursor limitation imposed by the same stomatal-closure-driven carbon shortage that depleted the sugar pool. Consistent with this, their fall paralleled the concurrent decline in the TCA-cycle intermediates citric and succinic acid (Figure 5B; Supplementary Table S3; Supplementary Figure S4), the carbon skeletons from which they are derived [27,72]. In contrast, the accumulating amino acids originate largely from accelerated protein turnover, since under carbon limitation, amino acids released by protein breakdown are routed into mitochondrial catabolism as alternative respiratory substrates that donate electrons to the transport chain to sustain residual ATP synthesis, while asparagine serves as the principal sink for liberated nitrogen [72]. Arbutin was likewise among the most severely depleted metabolites under drought (Supplementary Table S3). This collapse suggests a crucial role for stachyose as a glycosylated reserve, whose mobilization through hydrolysis both replenishes glucose to the carbon-starved root pool and releases a hydroquinone aglycone that can scavenge drought-induced ROS [73]. More broadly, the reliance on non-enzymatic antioxidant pools observed here echoes reports that phenolic accumulation in quinoa during stress serves to mitigate photo-oxidative damage when the photosynthetic rate is curtailed [38].

After rewatering, the root metabolome did not rebound uniformly but was reorganized selectively. Its most striking feature was the behavior of GABA and L-alanine, the two solutes most severely suppressed by drought, which not only recovered but also overshot control levels and emerged as the most dynamic nodes of the stress and recovery response of the root system. Among carbohydrates, only D-glucose returned to control, whereas D-fructose, D-xylose, D-ribose, and sucrose remained at drought-like levels (Figure 5B; Supplementary Figure S4); therefore, the restoration of root water status and Lp_rOS_ (Figure 2 and Figure 3) cannot be explained by a broad return of osmotically active sugars. We did not measure respiration or ATP directly; however, the concurrent normalization of the TCA-cycle intermediates citric and succinic acid is consistent with a recovered respiratory and ATP-generating capacity, the energetic currency required to repower aquaporin-mediated and active radial water transport and to reestablish Lp_rOS_ (Supplementary Table S3; Supplementary Figure S4; [15,72]). In brief, we present this as an interpretative framework rather than a demonstrated mechanism. In this view, the root regains hydraulic competence not by rebuilding its bulk osmotic but by restoring the metabolic machinery that sustains water transport, consistent with the rapid and near-complete recovery of Lp_rOS_ despite only partial carbohydrate replenishment. To date, these metabolites have been read only as indicators of belowground carbon and energy status. Establishing whether their recovery is also coupled to persistent stomatal restraint, and therefore whether they are the root-derived metabolomic cues posited in our hypothesis, requires that the root system metabolome be analyzed together with the physiological time course.

**Figure 7:**
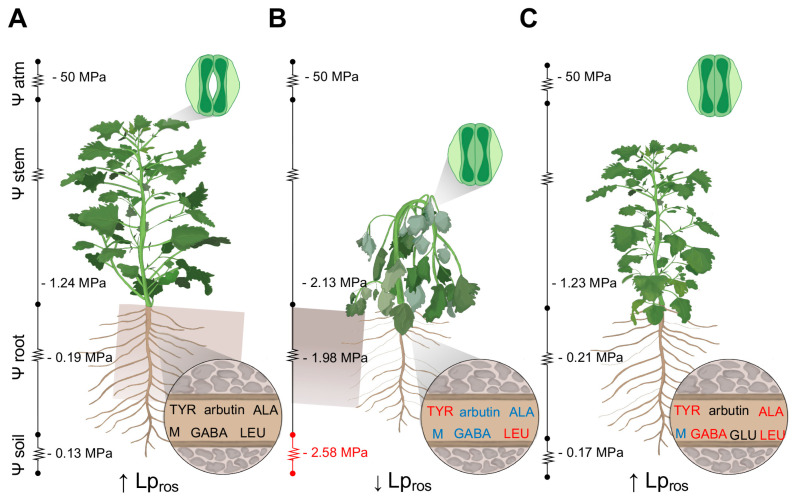
Schematic representation of belowground hydraulic and metabolomic recovery preceding stomatal reopening in drought-stressed coastal quinoa AZ1. (**A**) Well-watered plants exhibit open stomata, high water potential values (Ψ_soil_, Ψ_root_, Ψ_stem_), high osmotic root hydraulic conductance (Lp_rOS_), and a baseline root system polar metabolome. (**B**) Drought and severe water stress induce drastic stomatal closure and a sharp drop in Ψ, creating a hydraulic bottleneck at the soil–root interface. Root Lp_rOS_ decreases (↓Lp_rOS_), accompanied by the accumulation of key root system stress-responsive metabolites (e.g., GABA, GLU, ALA, arbutin). (**C**), Recovery, following rewatering, root hydraulics (↑Lp_rOS_) and water potential (Ψ) increased, and the metabolome returns to pre-stress levels, despite stomata remaining largely closed.

To address this, we integrated the metabolome with the physiological time course using pairwise Pearson matrices (Figure 6). Under control conditions, root metabolites were only loosely associated with physiological descriptors (Figure 6A), consistent with a non-limiting homeostatic state in which solute pools and water transport vary largely on their own. Under drought, the correlation structure narrowed to a single dominant relationship, since Lp_rOS_ was positively correlated with the root sugar pool, while no other metabolite-to-physiology association reached significance (Figure 6B). This indicates that, under water deficit, the residual cell-to-cell conductance scales primarily with the availability of soluble sugars, which directly reinforces the bioenergetic reading advanced above, namely that radial water transport is gated by the carbon and energy status of the root system rather than by bulk osmotic build-up. The decisive signature emerged upon rewatering, where root metabolites correlated strongly and positively with root water potential and, at the same time, negatively with stomatal conductance (Figure 6C and Figure 7). In other words, as solutes such as GABA and L-alanine rebounded, they tracked the rehydration of the root tissue itself, while g_s_ remained suppressed. This contrast is the within-treatment statistical signature of belowground and aboveground decoupling on which we base our interpretation, since the root metabolome followed the recovery of root water status rather than the resumption of stomatal function. Given the established role of GABA as a stress-responsive signaling metabolite that integrates carbon, nitrogen, and redox status and modulates membrane transport, its trajectory suggests that it is a candidate node among the residual root-derived cues hypothesized to constrain stomatal reopening ([59,73,74]; Figure 7). The prominence of nitrogen-rich solutes in the recovering root metabolome is consistent with earlier work on Chilean lowland quinoa, in which nitrogen-containing compounds, namely proline and polyamines, were the metabolites that best discriminated genotypes differing in stress tolerance, with the most tolerant coastal-central accession showing the largest proline accumulation and the most sensitive southern accession showing the weakest polyamine adjustment [37]. Our results extend this notion from shoot-based responses during stress imposition to the root system during stress relief, indicating that the nitrogen-rich fraction of the quinoa metabolome can be mobilized in both phases. We emphasize that these metabolite–physiology relationships are correlative, where functional validation, for instance, through exogenous application of candidate metabolites or genetic manipulation, will be required before a causal role can be established.

## 5. Conclusions

To our knowledge, this is the first account of post-drought recovery dynamics at the root level in the Chilean coastal ecotype *Chenopodium quinoa* AZ1, a phase that has remained unexplored in this species despite its recognized stress resilience. Our data reveal that recovery in quinoa follows a pronounced root-to-shoot temporal hierarchy, since within 24 h of rewatering, the root re-established water potential across the soil–plant–atmosphere continuum and restored Lp_rOS_ to near-control values despite persistent endodermal and xylem suberization, whereas stomatal conductance remained indistinguishable from drought-stressed plants. The second novel finding is the nature of this hydraulic recovery, because the root regained water-transport capacity not by rebuilding its osmotic sugar pool, which remained only partially replenished, but by reactivating its energy metabolism, as indicated by the normalization of TCA-cycle intermediates. This points to an energy-dependent, membrane-controlled transport route that bypasses the still-suberized apoplastic barriers of the roots. Finally, the recovering metabolome, led by GABA and L-alanine, which overshot the control while tracking root water status yet remained inversely related to stomatal conductance, suggests that nitrogen-rich signaling solutes may act as a previously unrecognized layer linking belowground rehydration to delayed stomatal reopening. Together, these findings reframe the quinoa root as a compartment that facilitates drought recovery. Establishing causality will require resolving whether this asymmetry reflects active root-derived signaling or a leaf-autonomous hydraulic–hormonal set-point through the quantification of ABA and aquaporins and reciprocal root and shoot manipulation. Establishing whether the root-paced recovery described here is a general property of the species or a specific attribute of coastal lowland germplasm will require a parallel analysis of salares and highland ecotypes, which differ from coastal accessions in root architecture, stress metabolism, and the balance between tolerance and productivity.

## Data Availability

The original contributions presented in this study are included in the article/Supplementary Materials. Further inquiries can be directed to the corresponding authors.

## Supplementary Materials

The following supporting information can be downloaded at: https://www.mdpi.com/article/10.3390/plants15152280/s1, Figure S1. Representative relationship between osmotic pressure gradient and volumetric flow rate (Q) in a single coastal quinoa AZ1 plant. Figure S2. Leaf (A), stem (B), and root system (C) water potential of coastal quinoa AZ1 plants. Figure S3. Representative images of coastal quinoa AZ1 under control, drought and recovery conditions. Figure S4. Quantitative analysis of 17 selected metabolites in roots of coastal quinoa AZ1. Table S1. Gas exchange parameters (g_s_ and E) and leaf vapor pressure deficit (VPD_leaf_) in response to drought and rewatering (*n* = 5). Table S2. Relative water content (RWC) of whole-plant, shoot, and root of coastal quinoa AZ1 subjected to drought and rewatering (*n* = 5). Table S3. Significant metabolites in roots of coastal quinoa AZ1 under control, drought, and recovery conditions.
