# Supplementary material for: Root Hydraulic and Metabolomic Recovery Outpaces Stomatal Reopening in Rewatered Quinoa
Source: Plants (Basel). 2026 Jul 25;15(15):2280. doi: 10.3390/plants15152280 (PMC13467730; doi:10.3390/plants15152280)
Supplement: Supplementary file 1 [file plants-15-02280-s001.zip › plants-4418502-supplementary.pdf]

**Supporting Information**

Article title: **Belowground recovery precedes stomatal reopening in drought-stressed quinoa: hydraulic and metabolomic evidence**
Authors: Flavia Dorochesi[1], Cesar Barrientos-Sanhueza[1]*, Marcos Roldán-Lazo[1], Romina Pedreschi[1], and Italo F. Cuneo[1]*
The following Supporting Information is available for this article:

**Supplementary Figure S1.** Representative relationship between osmotic pressure gradient and volumetric flow rate (Q) in a single coastal quinoa AZ1 plant. Each data point represents the mean Q measured at a given osmotic pressure gradient (0, -0.125 and -0.25 MPa), presented as mean ± SE (n = 5). The slope of the linear regression (solid line) was used to calculate osmotic root hydraulic conductance ($Lp_{ros}$).

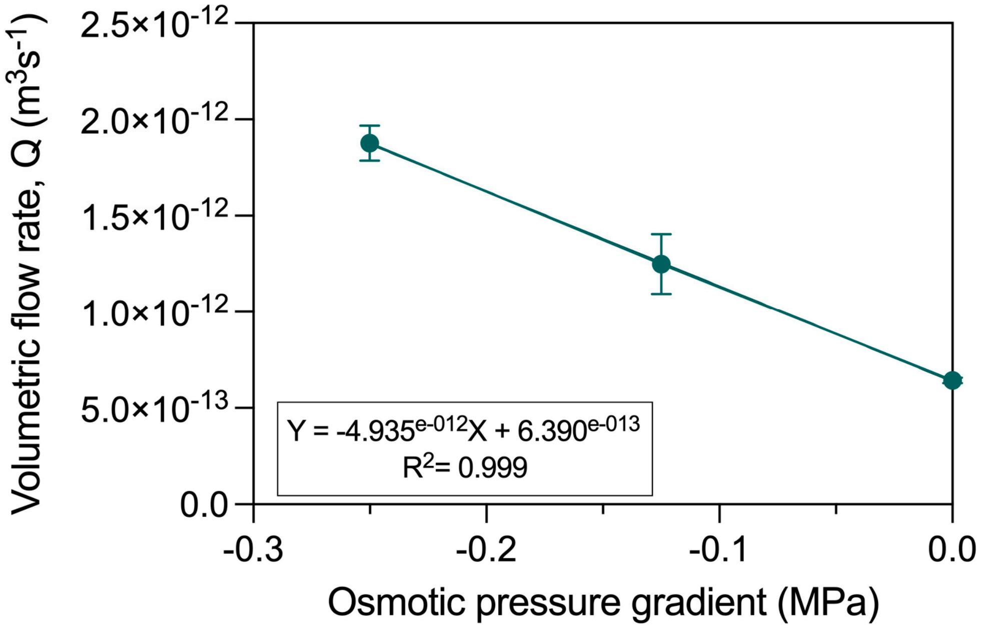

**Supplementary Figure S2.** Leaf (A), stem (B), and root system (C) water potential of coastal quinoa AZ1 plants. Measurements were taken under control, drought, and recovery (24 h after rewatering) conditions using Scholander pressure chamber. Values represent means ± SE ($n = 5$). Different letters indicate significant differences among treatments according to Tukey’s HSD test ($p < 0.05$).

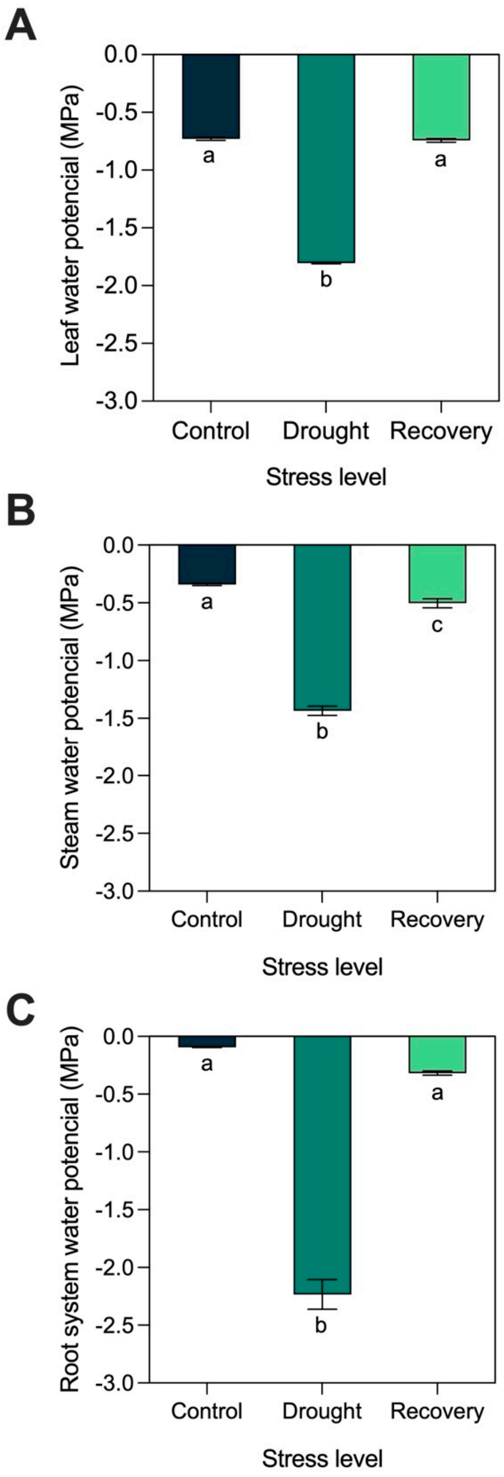
A
Leaf water potencial (MPa)
0.0
-0.5
-1.0
-1.5
-2.0
-2.5
-3.0
a
b
a
Control
Drought
Recovery
Stress level
B
Steam water potencial (MPa)
0.0
-0.5
-1.0
-1.5
-2.0
-2.5
-3.0
a
b
c
Control
Drought
Recovery
Stress level
C
Root system water potencial (MPa)
0.0
-0.5
-1.0
-1.5
-2.0
-2.5
-3.0
a
b
a
Control
Drought
Recovery
Stress level

**Supplementary Figure S3.** Representative images of coastal quinoa AZ1 under control, drought and recovery conditions. Drought-treated plants showed evident water loss and reduced turgor, whereas recovery plants displayed improved turgidity after rewatering.

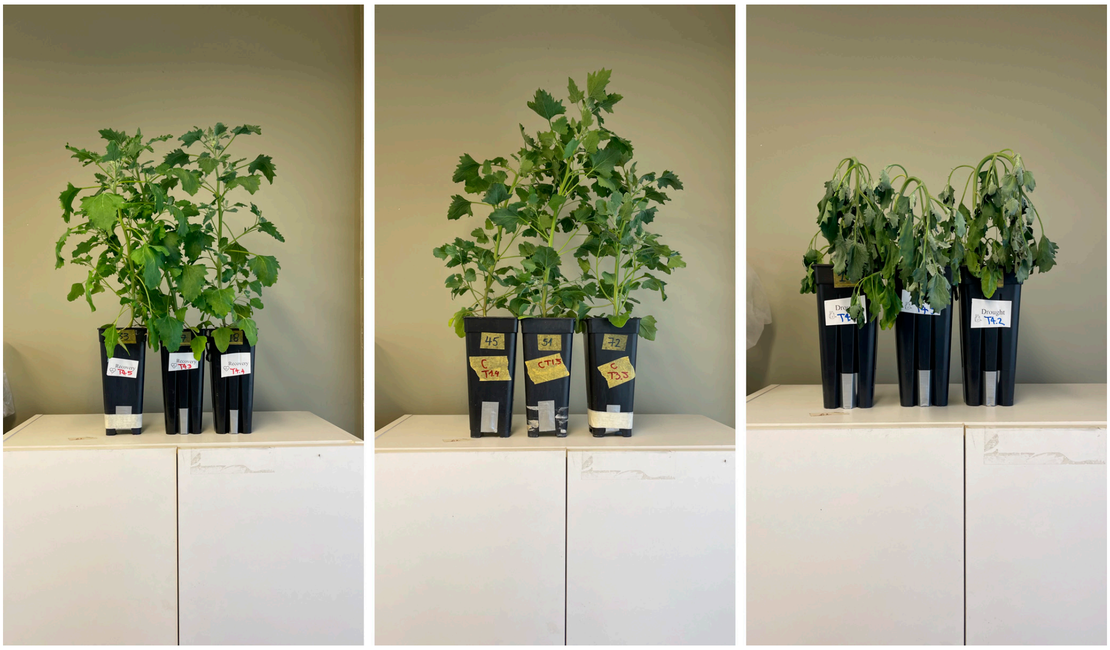

**Supplementary Figure S4.** Quantitative analysis of 17 selected metabolites in roots of coastal quinoa AZ1. Boxplots display normalized metabolite concentrations of sugars, organic acids, and amino acids under control, drought, and recovery conditions (n = 5); individual circles represent the values for each biological replicate. Data are presented as median and minimum and maximum values, with individual data points overlaid. Different letters above each treatment indicate statistically significant differences among groups according to Tukey's HSD test ($p < 0.05$).

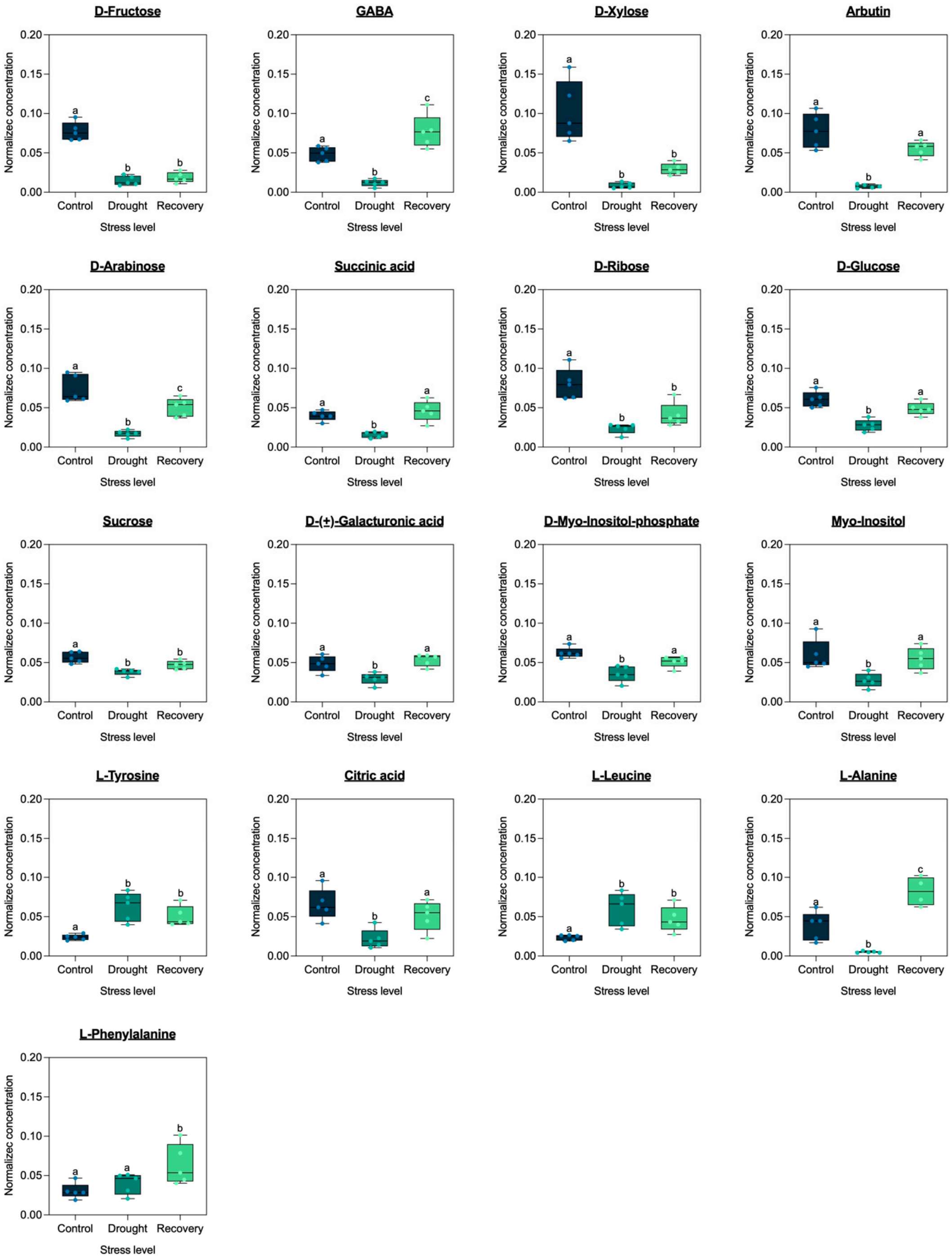
D-Fructose
GABA
D-Xylose
Arbutin
D-Arabinose
Succinic acid
D-Ribose
D-Glucose
Sucrose
D-(+)-Galacturonic acid
D-Myo-Inositol-phosphate
Myo-Inositol
L-Tyrosine
Citric acid
L-Leucine
L-Alanine
L-Phenylalanine
Normalizec concentration
0.00
0.05
0.10
0.15
0.20
Control
Drought
Recovery
Stress level

**Supplementary Table S1.** Gas exchange parameters ($g_s$ and E) and leaf vapor pressure deficit ($VPD_{leaf}$) in response to drought and rewatering (n = 5). Values are means ± SE. Different letters within a column indicate significant differences among treatments according to Tukey's HSD test ($p < 0.05$).

| Treatment | $g_{sw}$ (mol $m^{-2}$ $s^{-1}$) | E (mmol $m^{-2}$ $s^{-1}$) | VPD (KPa) |
|---|---|---|---|
| **Control** | 0.0324 ± 0.0063[a] | 0.5590 ± 0.1088[a] | 1.658 ± 0.046[a] |
| **Drought** | 0.0006 ± 0.0004[b] | 0.0080 ± 0.0071[b] | 1.522 ± 0.048[ab] |
| **Recovery** | 0.0132 ± 0.0039[b] | 0.1941 ± 0.0566[b] | 1.472 ± 0.041[b] |

**Supplementary Table S2.** Relative water content (RWC) of whole-plant, shoot, and root of coastal quinoa AZ1 subjected to drought and rewatering (n=5). Values are means ± SEM. Different letters within each column indicate significant differences among treatments according to Tukey's test ($p < 0.05$).

| Treatment | Total (%) | Shoot (%) | Root (%) |
|---|---|---|---|
| **Control** | 97.20 ± 0.89[a] | 97.16 ± 0.78[a] | 98.14 ± 0.67[a] |
| **Drought** | 80.13 ± 1.04[b] | 80.45 ± 0.94[b] | 79.43 ± 2.46[b] |
| **Recovery** | 96.52 ± 0.83[a] | 96.30 ± 0.54[a] | 95.52 ± 0.83[a] |

**Supplementary Table S3.** Significant metabolites in roots of coastal quinoa AZ1 under control, drought, and recovery conditions. Data are presented as mean ± SE ($n = 5$). Different letters within each row indicate significant differences among treatments (Tukey's HSD test, $p < 0.05$). Metabolite levels were normalized to the internal standard area and represent relative abundance. FDR: false discovery rate.

| Metabolite | Control (mean ± SD) | Drought (mean ± SD) | Recovery (mean ± SD) | ANOVA *p*-value | FDR |
|---|---|---|---|---|---|
| **β-Sitosterol** | 0.0557 ± 0.0106 [a] | 0.0252 ± 0.0095 [a] | 0.0467 ± 0.0100 [a] | 1.31E-03 | 4.60E-03 |
| **Unknown4** | 0.0525 ± 0.0090 [a] | 0.0273 ± 0.0110 [b] | 0.0440 ± 0.0073 [a] | 3.22E-03 | 8.30E-03 |
| **Unknown3** | 0.0407 ± 0.0076 [a] | 0.0198 ± 0.0073 [b] | 0.0754 ± 0.0251 [c] | 4.29E-04 | 1.91E-03 |
| **Unknown2** | 0.0457 ± 0.0227 [a] | 0.0245 ± 0.0073 [b] | 0.0589 ± 0.0110 [a] | 1.20E-02 | 2.02E-02 |
| **Sugar unknown3** | 0.0684 ± 0.0168 [a] | 0.0165 ± 0.0029 [b] | 0.0522 ± 0.0101 [a] | 3.61E-05 | 2.95E-04 |
| **Sugar unknown2** | 0.0686 ± 0.0120 [a] | 0.0155 ± 0.0053 [b] | 0.0553 ± 0.0071 [a] | 1.32E-02 | 3.23E-01 |
| **Sugar unknown1** | 0.0827 ± 0.0348 [a] | 0.0105 ± 0.0042 [b] | 0.0647 ± 0.0344 [a] | 4.42E-03 | 1.08E-02 |
| **Sucrose** | 0.0566 ± 0.0070 [a] | 0.0380 ± 0.0040 [b] | 0.0470 ± 0.0054 [b] | 7.90E-04 | 3.16E-03 |
| **Succinic acid** | 0.0398 ± 0.0063 [a] | 0.0160 ± 0.0038 [b] | 0.0459 ± 0.0129 [a] | 3.27E-04 | 1.60E-03 |
| **Stigmasterol** | 0.0510 ± 0.0093 [a] | 0.0295 ± 0.0102 [b] | 0.0506 ± 0.0065 [a] | 3.04E-03 | 8.27E-03 |
| **Oxalic acid** | 0.0620 ± 0.0364 [a] | 0.0150 ± 0.0046 [b] | 0.0554 ± 0.0116 [a] | 1.19E-02 | 2.02E-02 |
| **Myo-Inositol** | 0.0595 ± 0.0195 [a] | 0.0275 ± 0.0091 [b] | 0.0549 ± 0.0143 [a] | 1.11E-02 | 2.01E-02 |
| **Maltose** | 0.0560 ± 0.0188 [a] | 0.0272 ± 0.0054 [b] | 0.0500 ± 0.0104 [a] | 9.52E-03 | 1.90E-02 |
| **Malic acid** | 0.0573 ± 0.0115 [a] | 0.0405 ± 0.0074 [b] | 0.0400 ± 0.0069 [b] | 1.41E-02 | 2.30E-02 |
| **L-Valine** | 0.0260 ± 0.0095 [a] | 0.0516 ± 0.0178 [b] | 0.0454 ± 0.0078 [b] | 1.80E-02 | 2.85E-02 |
| **L-Tyrosine** | 0.0239 ± 0.0036 [a] | 0.0627 ± 0.0184 [b] | 0.0503 ± 0.0130 [b] | 1.73E-03 | 5.65E-03 |
| **L-Threonine** | 0.0280 ± 0.0063 [a] | 0.0506 ± 0.0195 [b] | 0.0581 ± 0.0196 [b] | 3.31E-02 | 4.63E-02 |
| **L-Phenylalanine** | 0.0304 ± 0.0101 [a] | 0.0398 ± 0.0133 [a] | 0.0637 ± 0.0257 [b] | 3.11E-02 | 4.48E-02 |
| **L-Leucine** | 0.0236 ± 0.0036 [a] | 0.0598 ± 0.0211 [b] | 0.0469 ± 0.0162 [b] | 9.70E-03 | 1.90E-02 |
| **L-Isoleucine** | 0.0231 ± 0.0066 [a] | 0.0583 ± 0.0226 [b] | 0.0510 ± 0.0200 [b] | 2.09E-02 | 3.20E-02 |
| **L-Alanine** | 0.0382 ± 0.0183 [a] | 0.0051 ± 0.0011 [b] | 0.0658 ± 0.0400 [c] | 3.84E-05 | 1.90E-04 |
| **GABA** | 0.0484 ± 0.0092 [a] | 0.0119 ± 0.0044 [b] | 0.0771 ± 0.0213 [c] | 2.68E-05 | 2.95E-04 |
| **Ergosta-7,22-dien-3-ol, (3β,22E)-,** | 0.0571 ± 0.0189 [a] | 0.0264 ± 0.0077 [b] | 0.0466 ± 0.0069 [a] | 6.41E-03 | 1.43E-02 |
| **Disaccharide unknown1** | 0.0721 ± 0.0250 [a] | 0.0190 ± 0.0069 [b] | 0.0444 ± 0.0173 [b] | 2.03E-03 | 5.91E-03 |
| **D-Xylose** | 0.1020 ± 0.0385 [a] | 0.0085 ± 0.0037 [b] | 0.0294 ± 0.0072 [b] | 7.34E-05 | 5.14E-04 |
| **D-Ribose** | 0.0801 ± 0.0199 [a] | 0.0236 ± 0.0065 [b] | 0.0408 ± 0.0152 [b] | 2.03E-04 | 1.12E-03 |
| **D-Myo-Inositol-phosphate** | 0.0624 ± 0.0068 [a] | 0.0356 ± 0.0101 [b] | 0.0513 ± 0.0072 [a] | 8.37E-04 | 3.16E-03 |
| **D-Glucose** | 0.0605 ± 0.0100 [a] | 0.0276 ± 0.0073 [b] | 0.0487 ± 0.0083 [a] | 2.05E-04 | 1.12E-03 |
| **D-Fructose** | 0.0771 ± 0.0119 [a] | 0.0145 ± 0.0060 [b] | 0.0186 ± 0.0066 [b] | 9.54E-04 | 4.67E-02 |
| **D-Arabinose** | 0.0741 ± 0.0174 [a] | 0.0171 ± 0.0042 [b] | 0.0506 ± 0.0116 [c] | 3.51E-05 | 2.95E-04 |
| **D-(+)-Galacturonic acid** | 0.0486 ± 0.0102 [a] | 0.0297 ± 0.0073 [b] | 0.0534 ± 0.0077 [a] | 2.05E-03 | 5.91E-03 |
| **Citric acid** | 0.0658 ± 0.0200 [a] | 0.0219 ± 0.0124 [b] | 0.0512 ± 0.0189 [a] | 5.58E-03 | 1.30E-02 |
| **Asparagine** | 0.0269 ± 0.0200 [a] | 0.0773 ± 0.0282 [b] | 0.0523 ± 0.0150 [a] | 1.10E-02 | 2.01E-02 |
| **Arbutin** | 0.0779 ± 0.0222 [a] | 0.0075 ± 0.0024 [b] | 0.0552 ± 0.0096 [a] | 1.36E-05 | 2.22E-04 |
| **2-Keto-l-gluconic acid** | 0.0597 ± 0.0153 [a] | 0.0313 ± 0.0178 [b] | 0.0489 ± 0.0086 [a] | 2.73E-02 | 4.05E-02 |
